# Hypereosinophilia With *Pseudomonas aeruginosa* Infection in Patients With Asthma on Anti-Interleukin-5 Biologics

**DOI:** 10.1016/j.chest.2026.02.017

**Published:** 2026-07-08

**Authors:** Rebecca Rivard, Samuel Mailhot-Larouche, Catherine Borg, Patel Pujan, Martin Klein, Philippe Lachapelle, Simon Couillard

**Affiliations:** a Faculté de médecine et des sciences de la santé, Université de Sherbrooke, Sherbrooke, QC, Canada; b University of Oxford, Oxford, England; c Royal Brompton and Harefield Hospitals, London, England

**Keywords:** anti-interleukin-5, anti-interleukin-5R, asthma, benralizumab, biologic, biomarkers, corticosteroids, eosinophils, Feno, infection, mepolizumab, *Pseudomonas aeruginosa*

## Abstract

Biologic therapies targeting IL-5 or its receptor reduce eosinophilia and exacerbations in asthma. Breakthrough eosinophilic attacks are usually considered biologic failure. We report 2 patients with severe asthma receiving mepolizumab or benralizumab who developed relapsing hypereosinophilia associated with *Pseudomonas aeruginosa* airway infection. Case 1 was a 71-year-old woman in Canada with severe eosinophilic asthma and COPD overlap whose eosinophils suddenly rebounded to 1,400 cells/μL during an acute exacerbation while on mepolizumab, coinciding with *P aeruginosa* infection and resolving after antibiotic treatment. Case 2 was a 56-year-old woman in the United Kingdom with late-onset asthma who developed marked eosinophil rebound (1,690 cells/μL) during benralizumab therapy, again associated with *Paeruginosa* infection and resolving after antipseudomonal therapy. These cases suggest that *P aeruginosa* may drive eosinophilia through IL-5-independent mechanisms. Sputum cultures should be considered during exacerbations in patients treated with anti-IL-5 or IL-5R therapies, regardless of type 2 biomarkers.

Asthma is a chronic inflammatory airway disease that can be severe and difficult to manage. Type 2 inflammation, including elevated blood eosinophil counts, is a key risk factor for exacerbations.^1^ Biologic therapies targeting IL-5 or its receptor reduce eosinophil counts and exacerbations in severe eosinophilic asthma.^2^ Breakthrough eosinophilic exacerbations are viewed as biologic treatment failures.^3^

We describe 2 patients with severe asthma treated with mepolizumab or benralizumab who developed relapsing eosinophilia in association with *Pseudomonas aeruginosa* airway infection (Fig 1). Written consent was obtained for both patients through appropriate ethics approvals.

## Case Presentations

### Case 1

In Canada, a 71-year-old woman who formerly smoked with severe eosinophilic asthma and COPD overlap was referred to the asthma clinic. She was receiving high-dose inhaled corticosteroids and long-acting beta-agonists, with a history of 1 exacerbation requiring oral corticosteroids over the previous year. Baseline lung function showed an FEV_1_ of 1.12 L (50% of predicted) with FEV_1_/FVC of 0.31. Her baseline exhaled nitric oxide (Feno) was 34 ppb, and her blood eosinophil count had ranged from 400 to 1,300 cells/μL in the previous year.

Investigations revealed a total IgE of 17,280 kU/L and positive Aspergillus-specific antibodies, consistent with allergic bronchopulmonary aspergillosis, and itraconazole was initiated. Parasitic infection, vasculitis, hypereosinophilic syndromes, and lymphoproliferative disease were excluded.

Mepolizumab therapy reduced blood eosinophils to 50 cells/μL with no exacerbations for 10 months. She later experienced an acute exacerbation requiring hospitalization despite adherence to mepolizumab and inhaled therapy, with eosinophils rising to 1,100 to 1,400 cells/μL. Sputum cultures grew *P aeruginosa*, and treatment with ciprofloxacin for 14 days and oral prednisolone for 5 days led to normalization of eosinophil counts (0-30 cells/μL).

### Case 2

In United Kingdom, a 56-year-old woman with late-onset asthma and comorbidities including nasal polyposis, recurrent sinusitis, and gastroesophageal reflux disease was seen by an asthma team. The patient experienced 5 to 6 hospitalizations annually for severe exacerbations in the last 2 years, despite high-dose inhaled corticosteroids/long-acting beta-agonists, and maintenance oral corticosteroids. At biologic initiation, her blood eosinophil count was 450 cells/μL (with prior peaks up to 2,120 cells/μL), FEV_1_ was 3.02 L (109% predicted), and Feno was 94 ppb.

Benralizumab therapy led to complete eosinophil depletion and oral corticosteroid discontinuation. Four months later, eosinophils rebounded to 1,690 cells/μL and Feno increased to 158 ppb despite adherence. Hematologic and immunologic investigation results were unremarkable, and CT pulmonary angiography showed mucus plugging and ground-glass opacities without bronchiectasis.

Sputum cultures grew *P aeruginosa*. She was treated with IV antibiotics and 14 days of ciprofloxacin while continuing benralizumab. Blood eosinophils normalized over the next 4 months (0-30 cells/μL), follow-up cultures were negative, and a CT scan the following year showed bronchial wall thickening with residual mucus plugging.

## Discussion

We report 2 patients from different countries who were receiving anti-IL-5/5R and who experienced hypereosinophilia in the context of *P aeruginosa* airway infection. In both cases, eosinophilia resolved after antipseudomonal treatment.

Although eosinophilic exacerbations have been reported with mepolizumab in the Inflammatory Profile of Exacerbations in Patients With Severe Refractory Eosinophilic Asthma Receiving Mepolizumab (MEX) study, these were typically noninfectious and associated with elevated Feno.^3^ In the Asthma Exacerbation Profile on Open Label Treatment With Benralizumab for Severe Eosinophilic Asthma (BenRex) study,^4^ most exacerbations under benralizumab were not associated with eosinophil elevation, consistent with the near-complete eosinophilic depletion occurring with benralizumab.^4^^,^^5^

In both cases described, *P aeruginosa* was isolated from airway cultures, suggesting that this organism may promote type 2 inflammation through non-IL-5 pathways. An association between *P aeruginosa* and eosinophilic airway inflammation has previously been reported in patients with noncystic bronchiectasis.^6^ To our knowledge, this is the first report of *P aeruginosa*-associated relapsing eosinophilia in non-bronchiectatic severe asthma treated with anti-IL-5 or anti-IL-5R therapy.

Interleukin-5-independent pathways may drive eosinophilia. Elastase B, a key *P aeruginosa* virulence factor, has been shown to induce type 2 innate inflammation by activating airway epithelial cells, promoting mucin and thymic stromal lymphopoietin release, and triggering amphiregulin-dependent estimated glomerular filtration rate activation with subsequent eotaxin-mediated eosinophil recruitment.^7^ Eosinophil migration from bone marrow to blood is primarily IL-5 mediated, but it also can occur via C-C motif chemokine receptor 3 binding to eotaxin, indicating that eosinophils may enter the circulation even when IL-5 signaling is impaired.^8^ Other contributors include IL-3 and granulocyte-macrophage colony-stimulating factor, which are produced during bacterial infection and asthma and promote eosinophil survival and proliferation, potentially enhancing eosinophil accumulation independently of IL-5.^9^^,^^10^ These mechanisms could potentially contribute to eosinophilic inflammation observed in the patients receiving anti-IL-5/5R.

## Conclusion

**Figure 1:**
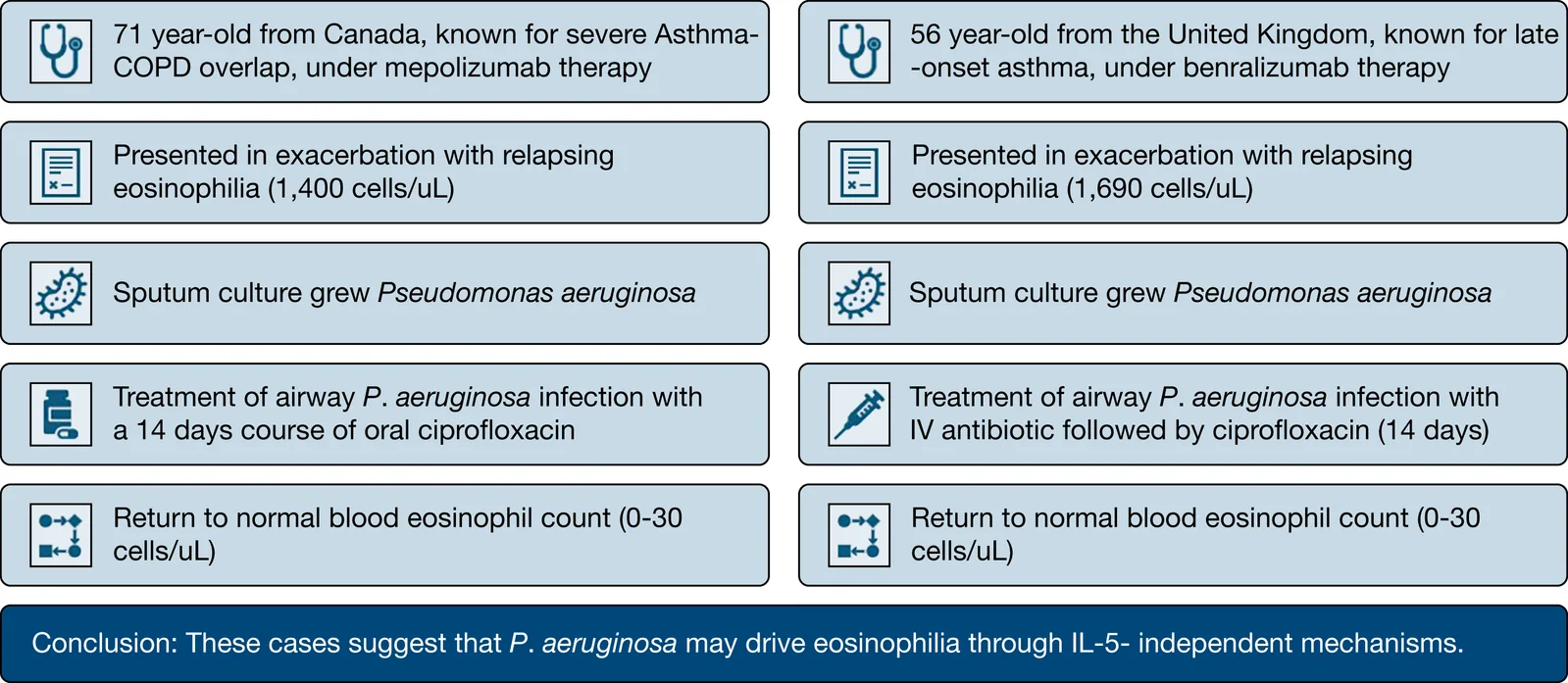
*Paeruginosa* under anti-IL5/5R therapy. IL = interleukin.

*Pseudomonas aeruginosa* infection was observed with hypereosinophilia in patients on anti-IL-5/5R. This highlights importance of sputum cultures when evaluating asthma exacerbations in patients treated with anti-IL-5 biologics independently of type 2 inflammatory biomarkers (Fig 1).

## Funding/Support

Supported by the 10.13039/501100020951Fonds de recherche du Québec—Santé and the Association Pulmonaire du Québec.

## Financial/Nonfinancial Disclosures

The authors have reported to *CHEST* the following: C. B. reports speaker honoraria from Chiesi, AstraZeneca and GlaxoSmithKline. P. P. has received speaker honoraria from AstraZeneca, Celltrion HealthCare, GlaxoSmithKline and Sanofi-Regeneron.; he has received sponsorship to attend/speak at international scientific meetings by/for AstraZeneca, Chiesi and Sanofi-Regeneron. P. L. reports speaker honoraria from AstraZeneca, Sanofi-Regeneron, GlaxoSmithKline, Boehringer Ingelheim, and Novartis outside of the submitted work and received consultancy fees from AstraZeneca, GlaxoSmithKline, and Sanofi-Regeneron, outside the submitted work. S. C. reports he has received nonrestricted research grants from the NIHR Oxford BRC, the Quebec Respiratory Health Research Network, the Association Pulmonaire du Québec, the Academy of Medical Sciences, AstraZeneca, bioMérieux, Circassia Niox Group, and Sanofi-Genyme-Regeneron; he is the holder of the Association Pulmonaire du Québec’s Research Chair in Respiratory Medicine and is a clinical research scholar of the Fonds de recherche du Québec; he received speaker honoraria from AstraZeneca, GlaxoSmithKline, Sanofi-Regeneron, Circassia Niox Group, and Valeo Pharma; he received consultancy fees for FirstThought, Apogee Therapeutics, Upstream Bio, AstraZeneca, GlaxoSmithKline, Sanofi-Regeneron, Access Biotechnology and Access Industries; he has received sponsorship to attend/speak at international scientific meetings by/for AstraZeneca and Sanofi-Regeneron. He is an advisory board member and detains stock options for Biometry Inc—a company developing a Feno device (myBiometry); he is co-inventor for the patent filed as “Method for alleviating dyspnea with neuromodulation.” He advised the Institut national d'excellence en santé et services sociaux (INESSS) for an update of the asthma general practice information booklet for general practitioners as well as therapeutic indications for Enerzair, and is a member of the asthma steering committee of the Canadian Thoracic Society. None declared (R. R., S. M. L., M. K.).
